# Supplementary material for: Nuclear Binding Protein 2/Nesfatin-1 Affects Trophoblast Cell Fusion during Placental Development via the EGFR-PLCG1-CAMK4 Pathway
Source: Int J Mol Sci. 2024 Feb 5;25(3):1925. doi: 10.3390/ijms25031925 (PMC10856506; doi:10.3390/ijms25031925)
Supplement: Supplementary file 1 [file ijms-25-01925-s001.zip › ijms-2805555-supplementary.pdf]

Nuclear Binding Protein 2/Nesfatin-1 Affects Trophoblast Cell Fusion during Placental Development via the EGFR-PLCG1-CAMK4 Pathway

Qinyu Dang [1], Yandi Zhu [1], Yadi Zhang [1], Zhuo Hu [1], Yuchen Wei [1], Zhaoyang Chen [1], Xinyin Jiang [2], Xiaxia Cai [1] and Huanling Yu [2,*]

[1] Department of Nutrition and Food Hygiene, School of Public Health, Capital Medical University, Beijing 100069, China; dang_qinyu@mail.ccmu.edu.cn (Q.D.); zhuyandi@ccmu.edu.cn (Y.Z.); yadizhang@mail.ccmu.edu.cn (Y.Z.); huzhuo99@mail.ccmu.edu.cn (Z.H.); weiyuchen_98@mail.ccmu.edu.cn (Y.W.); chenzhaoyang@mail.ccmu.edu.cn (Z.C.); caixx1988@ccmu.edu.cn (X.C.)

[2] Departments of Health and Nutrition Sciences, Brooklyn College of City University of New York, New York, NY 11210, USA; xinyinjiang@brooklyn.cuny.edu

* Correspondence: yuhlzjl@ccmu.edu.cn; Fax: +86-10-83911652

Supplementary documents

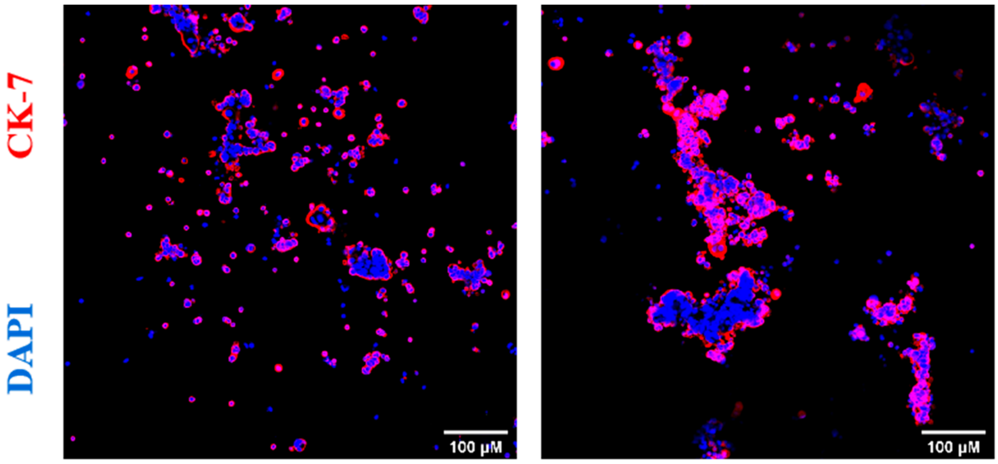

Figure S1, Identification of primary primary trophoblast cells. Immunofluorescence of cytokeratin-7 (CK-7) was used to identify isolated cell populations at the 24-hour time point. Cells were stained with anti-keratin-7 (red) or DAPI (blue). Scale bars represent 100 μm.

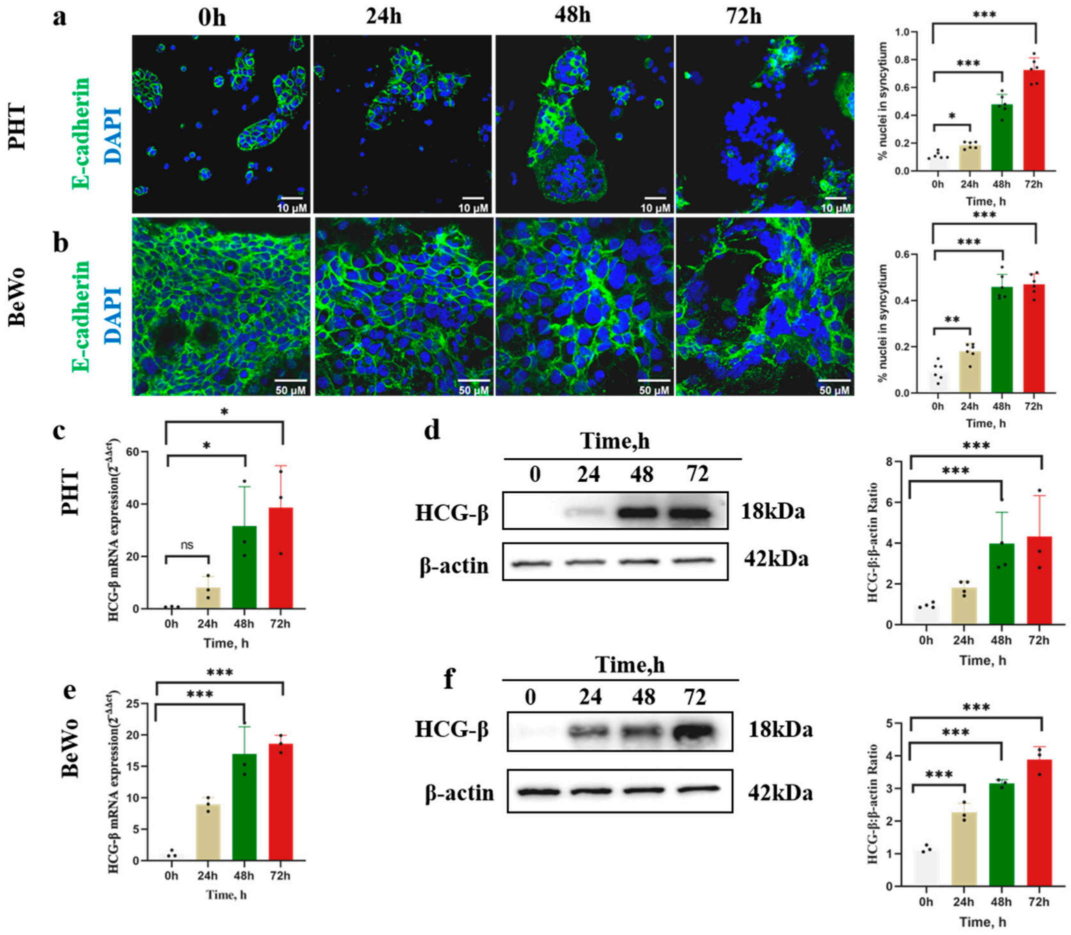

Figure S2，Increase in the degree of differentiation and fusion during trophoblast syncytization. (a) Representative immunofluorescence images depict changes in E-cadherin at different time points of primary trophoblast syncytialization. The scale lines are 20 μm and 50 μm, respectively. (b) Representative immunofluorescence images depict changes in E-cadherin at different time points of Forskolin-induced differentiation and fusion of BeWo cells. Cells were stained with anti-E-cadherin (green) or DAPI (blue). (c) mRNA levels of HCG-β at different time points in primary trophoblast syncytia; (d) Protein expression levels of HCG-β relative to β-actin at different time points in trophoblast syncytia. (e) HCG-β at different time points in Forskolin-induced differentiation and fusion of BeWo cells. mRNA levels; (f) Protein expression levels of HCG-β relative to β-actin at different time points of Forskolin-induced differentiation and fusion of BeWo cells. Data are expressed as mean ± SD and analyzed by one-way ANOVA and Tukey-Kramer multiple comparison test. (**p* < 0.05; ***p*<0.001; ****p*<0.001;).

a

b

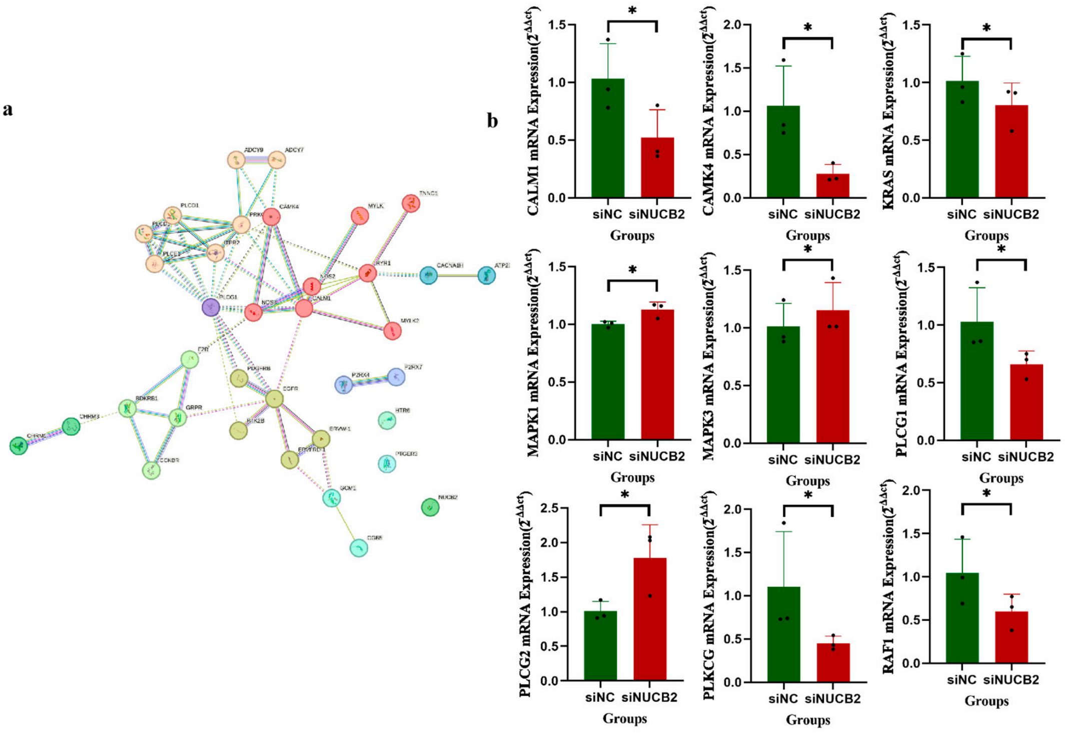

Figure S3. Genes involved in the calcium signaling pathway. **(a)** string database for protein interactions between calcium signaling pathway genes and trophectoderm differentiation fusion-related genes. **(b)** Expression of genes associated with EGFR gene expression in the calcium signaling pathway. Data are expressed as mean ± SD and analyzed by one-way ANOVA and Tukey-Kramer multiple comparison test.(**p*<0.05).

Table S1. Sequence of the primers for real-time PCR used in this study.

	Forward Primer	Reverse Primer
GAPDH(human)	GAGTCAACGGATTTGGTCGT	TTGATTTTGGAGGGATCTCG
β-actin(human)	AAAGCCACCCCACTTCTCTC	GCAATGCTATCACCTCCCCT
NUCB2(human)	GAGCCACACATTTAAAGTCTGAAGT	GCAGAGAAAAAGGAAGGGAGC
ERVW-1(human)	GCAACTGCTATCACTCTGCCACTC	AGACAGTGACTCCAAGTCCTCCAG
ERVFRD(human)	AGCAGCCGTAGTCCTTCAAA	AGGGGAAGAACCCAAGAGAA
GCM1(human)	GTCACCAACTTCTGGAGGCAC	GCTCTTCTTGCCTCAGCTTCTAA
β-HCG(human)	GCTTCAGTCCAGCACCTTTC	CACGGTGAAGTGACCTCAGA
β-actin(rat)	TGATGACATCAAGAAGGTGGTGAAG	TCCTTGGAGGCCATGTGGGCCAT
E-cadherin(rat)	CCTACAATGCTGCCATCGCCTAC	GGGTAACTCTCTCGGTCCAGTCC
NUCB2(rat)	CATGAAGACCACCCCAAAGT	TTCATCCAGGAATCCATCGT
EGFR(human)	GGTGAGTGGCTTGTCTGGAA	CCTTACGCCCTTCACTGTGT
PLCG1(human)	GGAAGACCTCACGGGACTTTG	GCGTTTTCAGGCGAAATTCCA
PLCG2(human)	CAGTCAAGGTTCTCGGTGCT	GCAGAAATGCCAGGTTTGGG
PRKCG(human)	CCTACGTGAACCCCGACTTC	CGTTGGGGACACCTAGTGG
KRAS(human)	AGGCCCTGTGTGAACCTTTG	CAAACTGCCCTAGTCCCTCC
RAF1(human)	AGATGCCGTGTTTGATGGCT	ACTGCACAGCACTCTGGTTG
MAPK1(human)	ACCAGACCTACTGCCAGAGAACC	TGGTCATTGCTGAGGTGTTGTGTC
MAPK3(human)	CATTGTGCAGGACCTGATGGAGAC	GTTGGCGGAGTGGATGTACTTGAG
CAMK4(human)	AGCCTCGTCCCGGATTACT	TAAGGCTTCTGGGTCCCCTT

Table S2. Antibodies used in this study.

Antibody	Host species	Catalogue number	Manufacturer	Final concentration
GAPDH	Mouse	G0100-100UL	LABLEAD	1：5000
β-HCG	Rabbit	ab53087	Abcam	1：500
GCM1	Mouse	ab236388	Abcam	1：2000
ERVW-1	Rabbit	ab179693	Abcam	1：500
Cytokeratin 7	Rabbit	ab181598	Abcam	1：100
E-Cadherin	Mouse	#14472	CST	1：50
NUCB2	Rabbit	ab229683	Abcam	1:500
β-actin	Rabbit	#4970	CST	1:5000
HERV-FRD	Rabbit	ab230235	Abcam	1:1000
EGFR	Rabbit	ab52894	Abcam	1:5000
P-EGFR	Rabbit	ab40815	Abcam	1:2500
PLCG1	Mouse	ab302940	Abcam	1:1000
P-PLCG1	Rabbit	ab76031	Abcam	1:100000
CAMK4	Rabbit	YT0627	immunoway	1:1000
P-CAMK4	Rabbit	YP0043	immunoway	1:1000

CST: Cell signaling technology, Danvers, MA, USA. Abcam: Abcam, Cambridge, UK. USA. Immunoway: immunoway Biotech, China.
